# A multi-angle analysis of injury induced by supplementation of soybean meal in *Litopenaeus vannamei* diets

**DOI:** 10.3389/frmbi.2023.1113635

**Published:** 2023-03-20

**Authors:** Kai Peng, Jianqiang Qiu, Chaozheng Li, Huijie Lu, Zhenxing Liu, Ding Liu, Wen Huang

**Affiliations:** ^1^ Collaborative Innovation Center of Aquatic Sciences, Institute of Animal Science, Guangdong Academy of Agricultural Sciences, Key Laboratory of Animal Nutrition and Feed Science in South China, Ministry of Agriculture and Rural Affairs, Guangdong Provincial Key Laboratory of Animal Breeding and Nutrition, Guangzhou, China; ^2^ College of Fisheries, Huazhong Agricultural University, Wuhan, China; ^3^ State Key Laboratory of Biocontrol, Institute of Aquatic Economic Animals and the Guangdong Province Key Laboratory for Aquatic Economic Animals, Sun Yat-Sen University, Guangzhou, China; ^4^ Institute of Animal Health, Guangdong Academy of Agricultural Sciences, Guangzhou, China; ^5^ Guangdong Provincial Engineering Research Center of Prawn Culture, Guangdong Havwii Agricultural Group Co., Ltd., Zhanjiang, China

**Keywords:** *Litopenaeus vannamei*, soybean meal, fish meal, hepatopancreas health, intestinal microbiota

## Abstract

Soybean meal is considered as one of the major components of *Litopenaeus vannamei* diets. However, most previous studies have focused on evaluating the effects of soybean meal on *L. vannamei* from the perspective of growth, physiology, and feed utilization; information regarding the analysis of serum metabolites, antioxidant and immune response, and intestinal microbiota is limited. Five diets were prepared, comprising 20% (T20), 28% (T28), 35% (T35), 42% (T42), and 50% (T50) soybean meal. A total of 600 shrimp were randomly distributed into 20 tanks (i.e., 30 shrimp per tank), with four tanks assigned to each dietary group. Shrimp were fed to apparent satiation during the 42-day feeding trial. The results showed that levels of serum globulin, alanine aminotransferase, and aspartate aminotransferase linearly increased (*p* < 0.01), but levels of high-density lipoprotein cholesterol linearly decreased (*p* < 0.001) as the proportion of soybean meal in the diet increased. Supplementation of shrimp diets with soybean meal linearly and quadratically increased (*p* < 0.05) serum total antioxidant capacity, levels of malondialdehyde, and activities of catalase, nitric oxide synthase and phenoloxidase. Hepatocytes in T35, T42, and T50 were shown to have different degrees of vacuolar degeneration, hepatic corpuscle atrophy, and star-like lumen loss. Dietary inclusion of soybean meal altered the composition of intestinal bacterial microbiota at phylum level, especially increasing the abundance of on other bacterial genera, whereas it had minimal impact on other bacterial genera and had no significant influence on the bacterial diversity. This study suggests that dietary supplementation of *L. vannamei* diets with soybean meal at concentrations exceeding 28% induces inflammation and oxidant damage of the hepatopancreas, and increases the risk of intestinal disease.

## Introduction

The scarcity and rising price of fish meal threaten the sustainable development of aquaculture. How to reduce the amount of fish meal in aquafeed has become an important question in this field. In recent years, soybean meal has been widely used to replace fish meal in both scientific research and commercial production, and has been presented as a promising alternative in aquafeed (Bae et al., 2020; Bruce et al., 2021; Wu et al., 2021a; Zhao et al., 2021a; Ding et al., 2022). Over the last decade, a number of studies have suggested that the replacement of fish meal with soybean meal is feasible when soybean meal is applied in appropriate doses (Yang et al., 2015; Kumar et al., 2017; Wang et al., 2020; Liu T. et al., 2021; Xu et al., 2021). However, dietary supplementation with soybean meal has also been reported to inhibit growth and induce damage in aquatic animals (Yun et al., 2017; Liu et al., 2018; Ding et al., 2019; Zhang et al., 2020; Liu Q. et al., 2021). Similarly, our recent study observed that diets exceeding 28% soybean meal significantly inhibited the growth, digestion, and muscle growth-related gene expression of shrimp (*Litopenaeus vannamei*) (Peng et al., 2022a). However, these previous studies mostly focused on evaluating the negative effects of soybean meal on aquatic animals from the perspective of growth, physiology, and feed utilization; studies examining multiple factors, such as serum metabolites, antioxidant and immune response, and intestinal microbiota, are rare. Studying the effect of soybean meal on these factors may contribute to further revealing the mechanism of action regarding the growth-inhibiting effect of soybean meal.

It is known that the growth of *L. vannamei* is highly dependent on fish meal (Suárez et al., 2009). Among the species used globally in aquaculture species, *L. vannamei* consumes the largest amount of fish meal, accounting for approximately 30% of total fish meal consumption in aquafeed (Gao et al., 2021). Therefore, research on the replacement of fish meal in *L. vannamei* is comparatively more important than in other species. Soybean meal is considered as one of the major components of shrimp diets (Shao, 2019). Based on previous studies of the application of soybean meal in *L. vannamei* diets (Amaya et al., 2007; Yang et al., 2015; Xu et al., 2021), our recent study indicated that the growth-inhibiting effect of high doses of dietary soybean meal on *L. vannamei* is attributed not only to the depression of feed digestibility but also to the inhibition of muscle growth (Peng et al., 2022a). Another study also reported that this growth-inhibiting effect may be due to damage of the hepatopancreas and intestine induced by inclusion of soybean meal in *L. vannamei* diets (Sun, 2015). This suggests that the influence of soybean meal on the health of *L. vannamei* manifests in many ways. This study was carried out to evaluate the effects of replacing fish meal with soybean meal on serum metabolites, antioxidants and the immune response, hepatopancreas histomorphology, and the intestinal bacterial microbiota of *L. vannamei*.

## Materials and methods

### Experimental diet preparation

Five experimental diets were prepared, comprising 20% (T20), 28% (T28), 35% (T35), 42% (T42), and 50% (T50) soybean meal. The ingredient and nutrient compositions of the diets are shown in **Table 1**. Diets were prepared and stored as previously detailed by Peng et al. (2022a). Briefly, all ingredients were ground to pass through a 320-μm sieve (AHZC1265 Hammer Mill; Buhler Machinery Co., Ltd., Guanghzou, China), mixed thoroughly (AHML2000 Mixer; Buhler Machinery Co., Ltd.), and then extruded into 2-mm pellets (SLX-80 Twin-screw Extruder; South China University of Technology Machinery Factory, Guangzhou, China). After being pelleted, the feeds were dried at 55°C for 12 h (HMO-205 Oven Dryer; Haiming Electronic Technology Co., Ltd., Dongguan, China) and stored at –20°C until use. Feeds were put into sealed bags and delivered to the fishery when needed.

**Table 1:** Ingredients and approximate compositions (g/kg DM) of experimental diets.

Item	Diet				
	T20	T28	T35	T42	T50
Ingredients					
Fishmeal (Peru, crude protein 68%)	250	200	150	100	50
Soybean meal (crude protein 46%)	200	280	350	420	500
Peanut bran	120	120	120	120	120
Chicken meal	100	100	100	100	100
Wheat flour	220	200	180	160	130
Fish oil	20	20	20	20	20
Soy lecithin	20	24	27	30	34
Monocalcium phosphate	15	15	15	15	15
Vitamin premix	2	2	2	2	2
Mineral premix	5	5	5	5	5
Lysine	0	0	0.5	1	1.5
Methionine	2	3	3.5	4.5	5.5
Choline chloride	2	2	2	2	2
Salt	3	3	3	3	3
Sodium alginate	12	12	12	12	12
Cellulose	29	14	10	5.5	0
Total	1000	1000	1000	1000	1000
Analyzed nutrient components					
Dry matter	928	929	928	926	927
Crude protein	414	420	418	420	416
Crude lipid	84	84	83	82	82
Ash	75	77	74	75	77
Lysine	23	23	23	23	23
Methionine	8.8	9.0	8.7	8.8	9.0

The proportion of soybean meal in each diet was 20% (T20), 28% (T28), 35% (T35), 42% (T42), or 50% (T50).

One kilogram of each diet provided: vitamin A 3,230 IU, vitamin D 1,600 IU, vitamin E 160 mg, vitamin K_3_ 4 mg, vitamin B_1_ 4 mg, vitamin B_2_ 8 mg, vitamin B_6_ 4.8 mg, vitamin B_12_ 0.016 mg, nicotinic acid 28 mg, pantothenic acid calcium 16 mg, biotin 0.064 mg, folic acid 1.285 mg, inositol 40 mg, Ca 1150 mg, K 180 mg, Mg 45 mg, Fe 50 mg, Zn 40 mg, Mn 9.5 mg, Cu 7.5 mg, Co 1.25 mg, I 0.16 mg, and Se 0.25 mg.

### Experimental procedure

The protocol (no. GAAS20210501) of this study was approved by the Animal Care and Use Committee of Guangdong Academy of Agricultural Sciences (Guangdong, China). The experiment was conducted in an indoor circulating water aquaculture system between September and October 2021.

A total of 600 shrimp (initial body weight of approximately 5.8 g) were randomly distributed into 20 tanks (i.e., 30 shrimp per tank), with four tanks being assigned to each diet. Shrimp were hand fed to apparent satiation (i.e., with feed approximately 4% of their body weight per day) three times a day at 08:00, 14:00, and 20:00. During the 42-day feeding trial, the water temperature was 25–27°C, the dissolved oxygen in the water was above 5.0 mg/L, the water pH was 7.6–8.0, the water salinity was 5‰–6‰, and ammonia nitrogen and nitrite concentrations in the water were below 0.01 mg/L.

### Sampling

Before sampling, all shrimp were fasted for 24 h. Blood was collected from the cardio-coelom of 15 shrimp in each tank, pooled, and kept at room temperature for 30 min, then centrifuged at 3,500 rev/min for 10 min. The resultant serum was stored at –80°C for the analysis of serum metabolites and antioxidant and immune parameters.

Three shrimp per tank were randomly selected for the histological examination of the hepatopancreas. Samples were fixed in 4% paraformaldehyde solution for 24 h. Slices were stained with the hematoxylin and eosin (Howard and Smith, 1983) and examined under light microscopy (Wang et al., 2021).

The intestines of three shrimp per tank were sampled on a clean bench, put into a 1.5-mL sterile centrifuge tube, and immediately stored at –80°C for subsequent DNA extraction.

### Analysis of nutrient compositions of diets

The nutrient compositions of the diets were analyzed using the methods of the AOAC (1999). Dry matter was measured by drying samples to a constant weight at 105°C (AOAC, #930.15). Crude protein was calculated by determining the total nitrogen (N × 6.25) using the Kjeldahl method (2300-Autoanalyzer, FOSS, Denmark) (AOAC, #2001.11). Crude lipid was measured by gravimetric analysis following ether extraction of lipids according to the Soxhlet method (36680-analyzer, BUCHI, Switzerland) (AOAC, #920.39). Ash was examined by combustion in a muffle furnace at 550°C for 6 h (AOAC, 1999; #942.05). Dietary lysine and methionine contents were determined by chromatography (Xu et al., 2016).

### Serum metabolites analysis

The following serum metabolites were measured using a BK-200VET biochemical analyzer (OLABO, China): albumin (ALB), globulin (GLOB), total cholesterol (TCHO), alanine aminotransferase (ALT), aspartate aminotransferase (AST), high-density lipoprotein cholesterol (HDLC), low-density lipoprotein cholesterol (LDLC), and glucose (GLU). The following serum antioxidant and immune parameters were determined by commercial kits provided by Nanjing Jiancheng Bioengineering Institute (Nanjing, China): total antioxidant capacity (TAOC; #A015-1), superoxide dismutase (SOD; #A001-1-2), catalase (CAT; #A007-1-1), glutathione peroxidase (GPx; #A005-1-2), malondialdehyde (MDA; #A003-1-2), alkaline phosphatase (AKP; #A059-2-2), lysozyme (LZM; #A050-1-1), nitric oxide synthase (NOS; #A014-2-2), and phenoloxidase (PPO; #A136-1-1).

### Intestinal bacterial communities analysis

The methods of bacterial DNA extraction and 16S rRNA sequencing followed the procedures described by Peng et al. (2021a). The V3–V4 region of 16S was targeted using primers 347F-CS1 (5′-ACACTGACGACATGGTTCTACAGGAGGCAGCAGTRRGGAAT-3′) and 803R-CS2 (5′-TACGGTAGCAGAGACTTGGTCTCTACCRGGGTATCTAATCC-3′). The real-time PCR conditions for 16S rRNA were 3 min at 95°C, followed by 45 cycles of 30 s at 95°C, 30 s at 60°C, and 45 s at 72°C. The composition and diversity of intestinal bacterial communities were analyzed using Illumina PE250 sequencing technology. Sequencing reads were assigned to each sample and analyzed with the QIIME package. Alpha diversity parameters were calculated using the Phyloseq Package (McMurdie and Holmes, 2013). Beta diversity was analyzed using the Pheatmap Package (Kolde and Kolde, 2015). Phenotypic classification prediction (pathogenic) of bacterial communities was conducted using BugBase, based on the Greengenes database (Ward et al., 2017). The 16S sequencing raw sequence reads were deposited in the NCBI sequence read archive (SRA) database, with the BioProject accession number PRJNA911431.

### Statistical analysis

IBM SPSS Statistics version 17.0 software (IBM Corporation, Armonk, NY, USA) was used to analyze the experimental data. All data were compared through one-way ANOVA, with the tank as the statistical unit and treatment as a fixed effect. Polynomial contrasts were used to test linear and quadratic responses to dietary soybean meal levels. The level of significance was set at a *p*-value < 0.05.

## Results

### Serum metabolites

The levels of GLOB linearly increased (*p* < 0.01) as dietary soybean meal increased from 200 to 500 g/kg and the increase reached significance at 280, 350, 420, and 500 g/kg (**Table 2**). Levels of ALT and AST linearly increased (*p* < 0.001), whereas HDLC levels linearly decreased (*p* < 0.001), as dietary soybean meal increased. All shrimp had similar (*p* > 0.05) levels of serum ALB, TCHO, LDLC, and GLU across the groups.

**Table 2:** Serum metabolite concentrations of *L. vannamei* fed experimental diets.

Item	Diet					SEM	*p*-value		
	T20	T28	T35	T42	T50		*P*	*L*	*Q*
ALB (g/L)	39.61	40.49	39.30	37.27	40.22	2.36	0.713	0.720	0.621
GLOB (g/L)	30.76^b^	37.35^a^	38.25^a^	38.55^a^	42.08^a^	2.52	< 0.01	< 0.01	0.315
TCHO (mmol/L)	1.03	1.17	1.12	1.04	1.10	0.22	0.964	0.987	0.766
ALT (U/L)	89.63^d^	111.66^c^	140.33^b^	164.32^a^	180.21^a^	8.54	< 0.001	< 0.001	0.502
AST (U/L)	78.63^c^	87.31^bc^	91.15^bc^	95.78^b^	116.40^a^	5.60	< 0.001	< 0.001	0.138
HDLC (mmol/L)	0.35^a^	0.24^b^	0.25^b^	0.23^b^	0.15^c^	0.02	< 0.001	< 0.001	0.849
LDLC (mmol/L)	0.05	0.04	0.04	0.05	0.05	0.01	0.600	0.393	0.940
GLU (g/L)	3.75	3.77	3.69	4.05	3.87	0.31	0.787	0.465	0.962

The proportion of soybean meal in each diet was 20% (T20), 28% (T28), 35% (T35), 42% (T42), or 50% (T50).

ALB, albumin; GLOB, globulin; TCHO, total cholesterol; ALT, alanine aminotransferase; AST, aspartate aminotransferase; HDLC, high-density lipoprotein cholesterol; LDLC, low-density lipoprotein cholesterol; GLU, glucose.

SEM, standard error of the mean; P, overall effect; L, linear effect; Q, quadratic effect.

Different letters within a row indicate a significant difference (p < 0.05).

### Serum antioxidant and immune response

Activities of serum TAOC and CAT, and levels of MDA linearly (*p* < 0.001) and quadratically (*p* ≤ 0.001) increased as dietary soybean meal increased from 200 to 500 g/kg, and the increase reached significance at 350, 420, and 350 g/kg, respectively (**Table 3**). The activities of NOS linearly (*p* < 0.001) and quadratically (*p* < 0.05) increased as dietary soybean meal increased, and the increase reached significance at 280, 350, 420, and 500 g/kg. PPO activities linearly (*p* < 0.001) increased as dietary soybean meal increased, and the increase reached significance at 350, 420, and 500 g/kg. Dietary treatments did not alter (*p* > 0.05) the serum activities of SOD, GPx, AKP, and LZM.

**Table 3:** Serum antioxidant and immune response of *L. vannamei* fed experimental diets.

Items	Diet					SEM	*p*-value		
	T20	T28	T35	T42	T50		*P*	*L*	*Q*
Antioxidant parameter									
TAOC (U/mL)	0.60^d^	0.64^d^	0.76^c^	0.86^b^	1.28^a^	0.04	< 0.001	< 0.001	< 0.001
SOD (U/mL)	64.77	68.45	65.12	67.61	65.36	2.64	0.613	0.958	0.432
CAT (U/mL)	13.18^c^	13.03^c^	15.30^c^	22.46^b^	30.42^a^	1.68	< 0.001	< 0.001	< 0.001
GPx (U/mL)	39.34	38.48	39.57	37.89	39.49	3.15	0.973	0.968	0.790
MDA (nmol/mL)	1.75^d^	1.79^d^	2.84^c^	3.90^b^	5.72^a^	0.37	< 0.001	< 0.001	0.001
Immune parameter									
AKP (U/mL)	2.22	2.31	2.48	2.40	2.38	0.17	0.692	0.336	0.355
LZM (U/mL)	143.81	145.51	142.45	145.33	143.36	9.84	0.996	0.976	0.973
NOS (U/mL)	22.80^b^	35.77^a^	35.95^a^	37.63^a^	40.93^a^	2.28	< 0.001	< 0.001	0.027
PPO (U/mL)	1.46^b^	1.90^b^	3.84^a^	4.03^a^	4.43^a^	0.45	< 0.001	< 0.001	0.123

The proportion of soybean meal in each diet was 20% (T20), 28% (T28), 35% (T35), 42% (T42), or 50% (T50).

TAOC, total antioxidant capacity; SOD, superoxide dismutase; CAT, catalase; GPx, glutathione peroxidase; MDA, malondialdehyde; AKP, alkaline phosphatase; LZM, lysozyme; NOS, nitric oxide synthase; PPO, phenoloxidase.

SEM, mean standard error; P, overall effect; L, linear effect; Q, quadratic effect.

Different letters within a row indicate a significant difference (p < 0.05).

### Hepatopancreas histological examination

**Figure 1:**
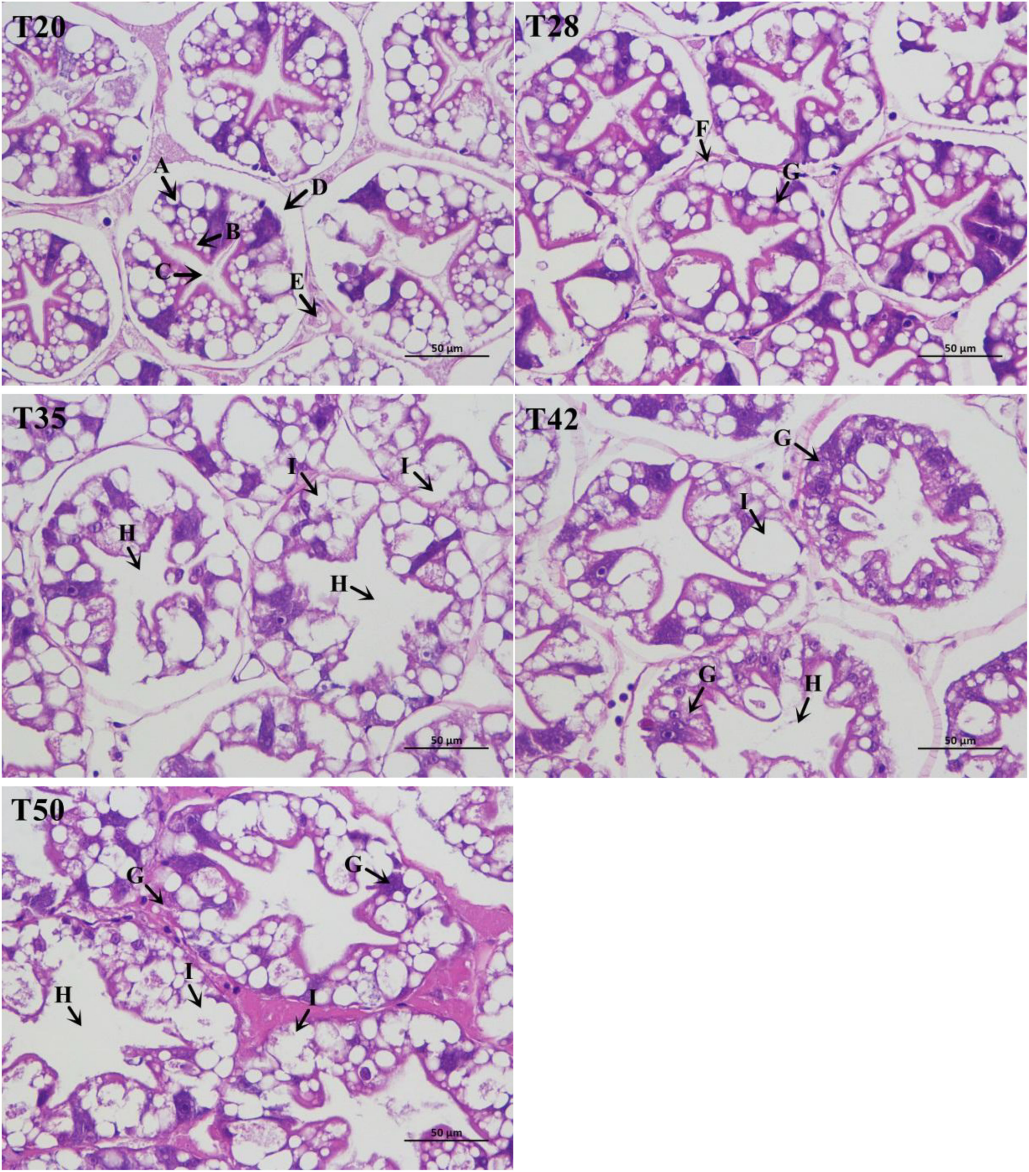
Histological appearance of the hepatopancreas (× 400) stained with hematoxylin and eosin. The proportion of soybean meal in each diet was 20% (T20), 28% (T28), 35% (T35), 42% (T42), or 50% (T50). **(A)** Absorptive cells; **(B)** secretory cells; **(C)** star shape of the lumen; **(D)** basal membrane; **(E)** tubules; **(F)** basal membrane atrophy; **(G)** inflammatory cell infiltration (unknown substances); **(H)** star-like lumen loss; and **(I)** vacuolar degeneration.

The morphology of shrimp hepatopancreas is shown in **Figure 1**. The structure of hepatocytes in shrimp fed the T20 and T28 diets was normal, with neatly arranged hepatic corpuscles, complete basement membranes, star-like lumens, and abundant absorption cells. However, hepatocytes in T35, T42, and T50 showed varying degrees of vacuolar degeneration, hepatic corpuscle atrophy, and star-like lumen loss. Clearly, degeneration of or damage to hepatocytes increased as dietary soybean meal increased from 350 to 500 g/kg.

### Intestinal bacterial microbiota

**Figure 2:**
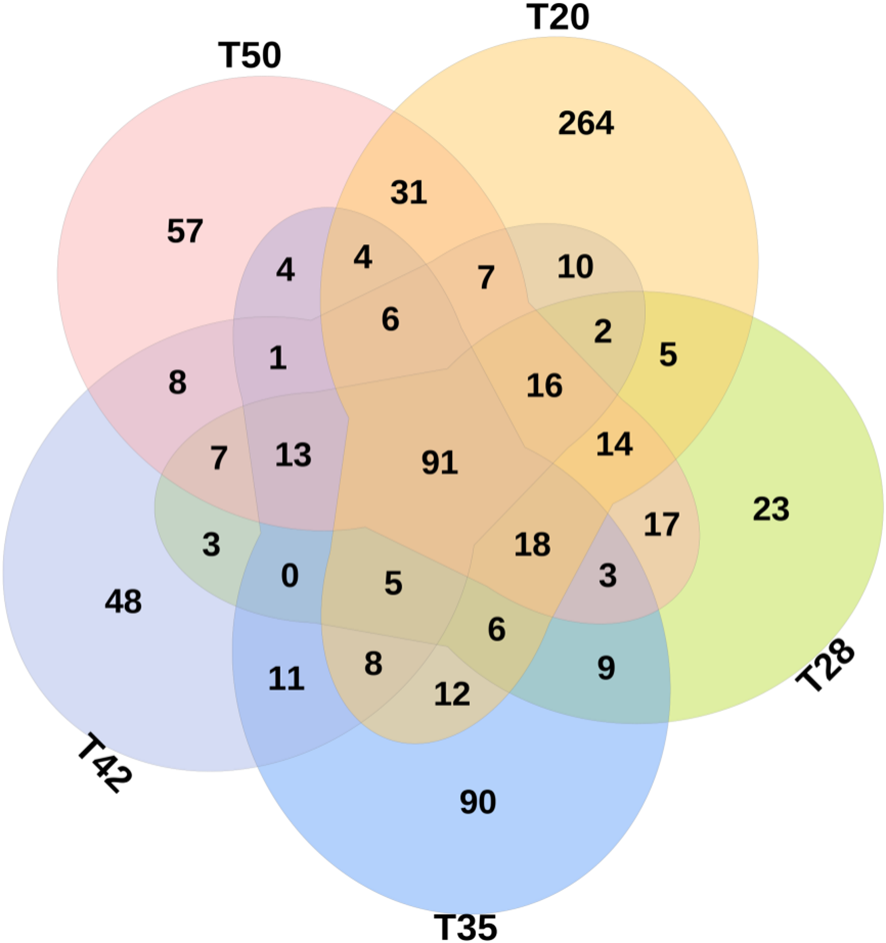
Venn diagrams showing unique and shared operational taxonomic units (OTUs) (at 97% similarity). Values are the numbers of OTUs calculated using the total data set at genus level. The proportion of soybean meal in each diet was 20% (T20), 28% (T28), 35% (T35), 42% (T42), or 50% (T50).

**Figure 3:**
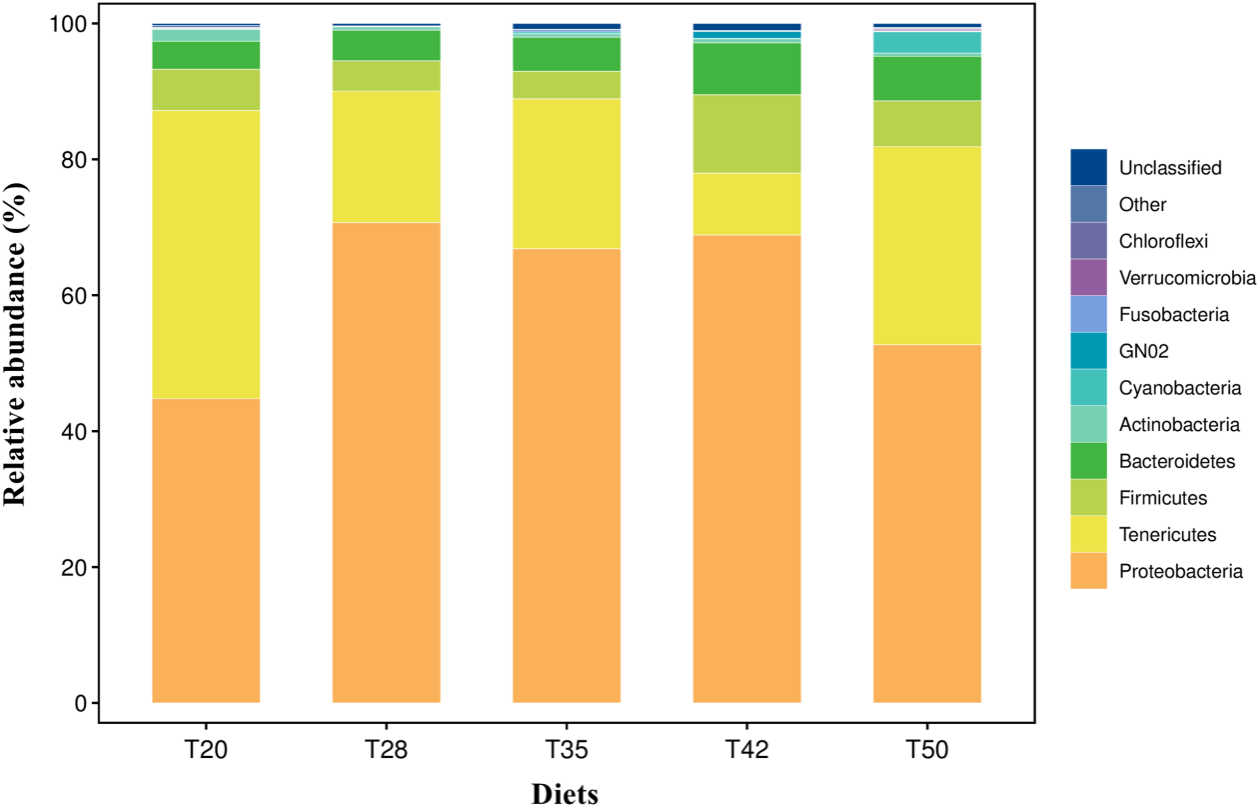
Taxonomic profile and relative abundance of the top 10 bacterial phyla. The proportion of soybean meal in each diet was 20% (T20), 28% (T28), 35% (T35), 42% (T42), or 50% (T50).

**Figure 4:**
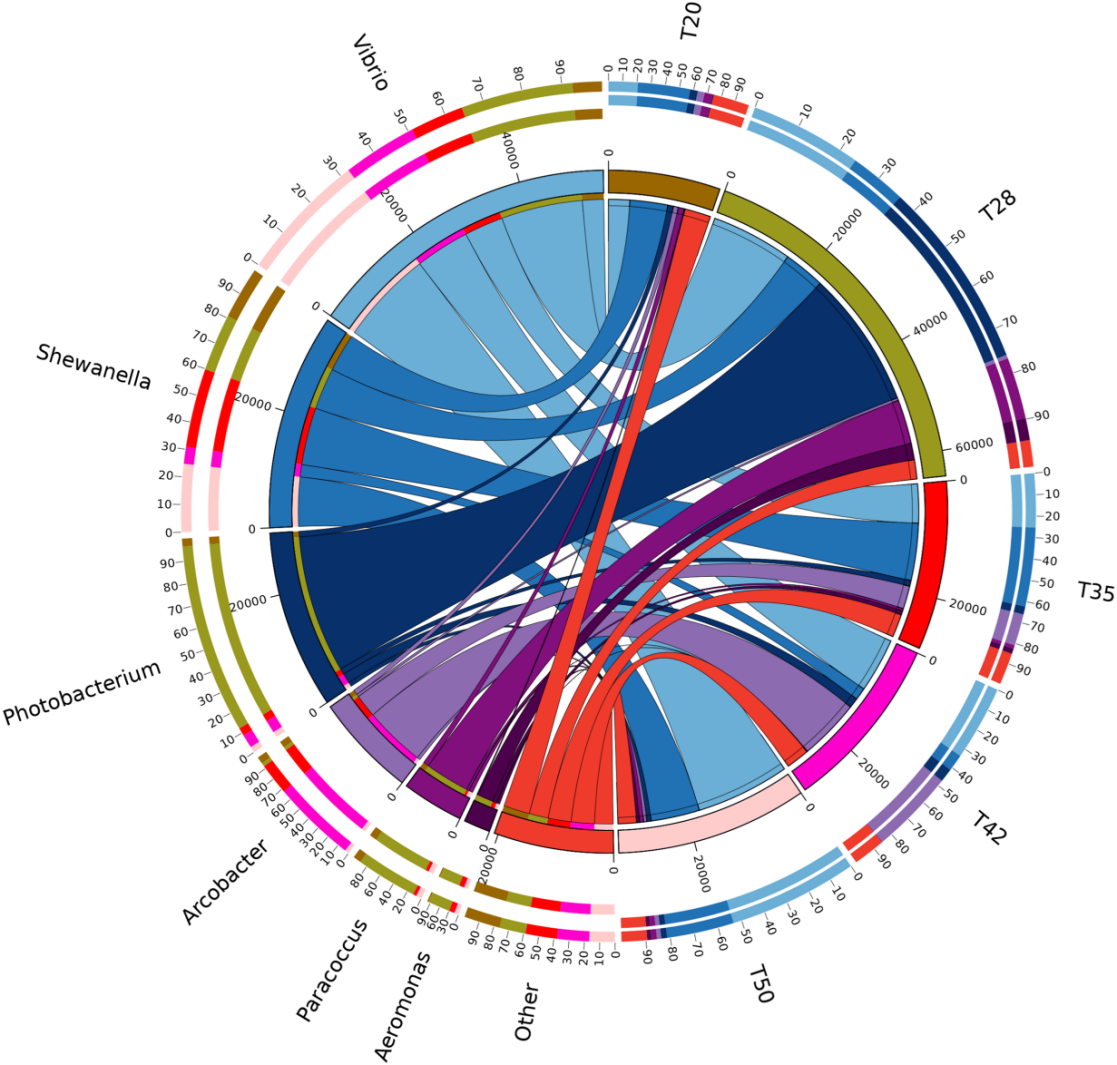
Circos map of the relationship between diets and relatively abundant bacterial genera (tags number > 2,000). The proportion of soybean meal in each diet was 20% (T20), 28% (T28), 35% (T35), 42% (T42), or 50% (T50).

**Figure 5:**
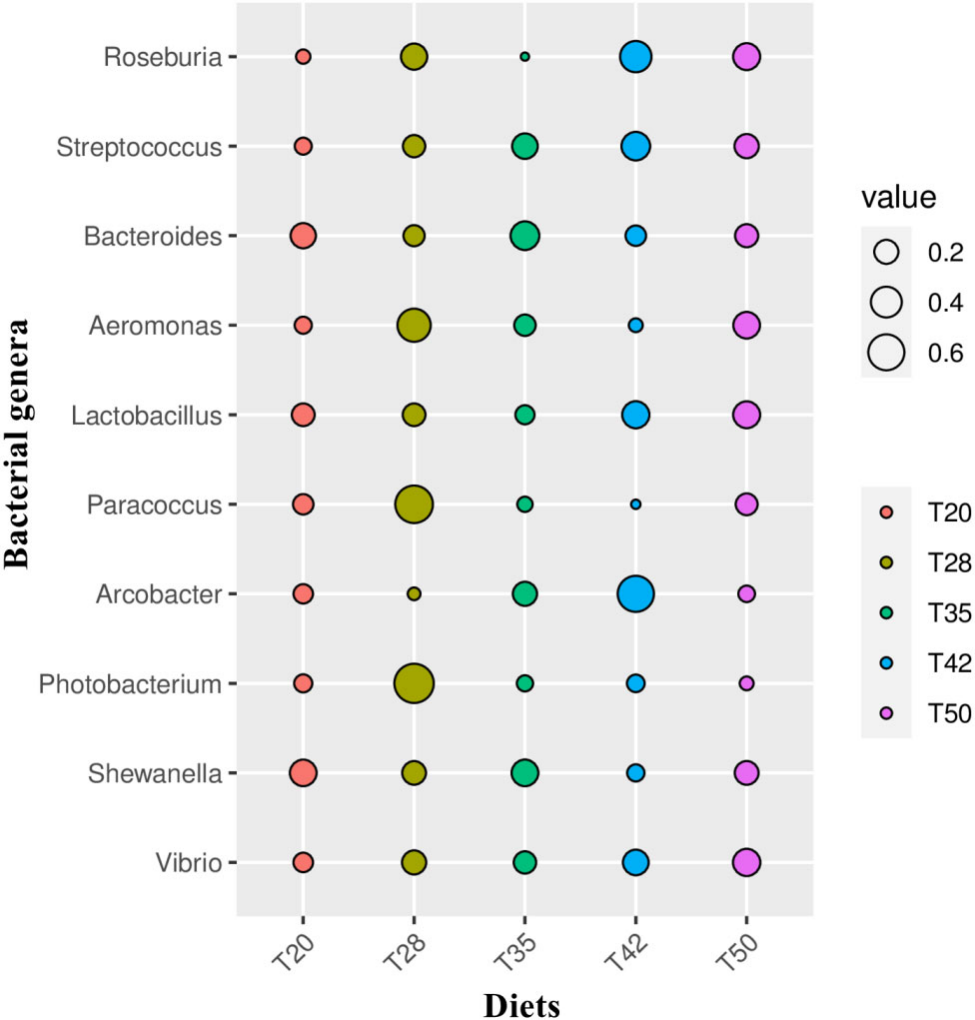
Relative abundances of the 10 most abundant bacterial genera. The indicator value of each bacterial genus in the comparison groups was calculated using the LABDSV package in R. Cross-validation is used for statistical verification to obtain the *p*-value. As shown in the bubble graph, the biomarkers of each group can be intuitively identified by the bubble size. The proportion of soybean meal in each diet was 20% (T20), 28% (T28), 35% (T35), 42% (T42), or 50% (T50).

The Venn diagram shows that 314 operational taxonomic units (OTUs) were shared by five groups (**Figure 2**). The number of unique OTUs in T20, T28, T35, T42, and T50 groups was 264, 23, 90, 48, and 57, respectively. The top 10 dominant bacterial phyla in the intestine of shrimp (shown in **Figure 3**) were Proteobacteria, Tenericutes, Firmicutes, Bacteroidetes, Actinobacteria, Cyanobacteria GN02, Fusobacteria, Verrucomicrobia, and Chloroflexi. Compared with the T20 group, the relative abundances of Proteobacteria and Bacteroidetes increased (*p* < 0.05), but the relative abundance of Tenericutes decreased (*p* < 0.05) in other groups. The Circos map of the relationship between the groups and relatively abundant bacterial genera (tag number > 2,000) is shown in **Figure 4**. Relative abundances of the top 10 abundant bacterial genera are shown in **Figure 5**, i.e., *Roseburia*, *Streptococcus*, *Bacteroides*, *Aeromonas*, *Lactobacillus*, *Paracoccus*, *Arcobacter*, *Photobacterium*, *Shewanella*, and *Vibrio*.

**Figure 6:**
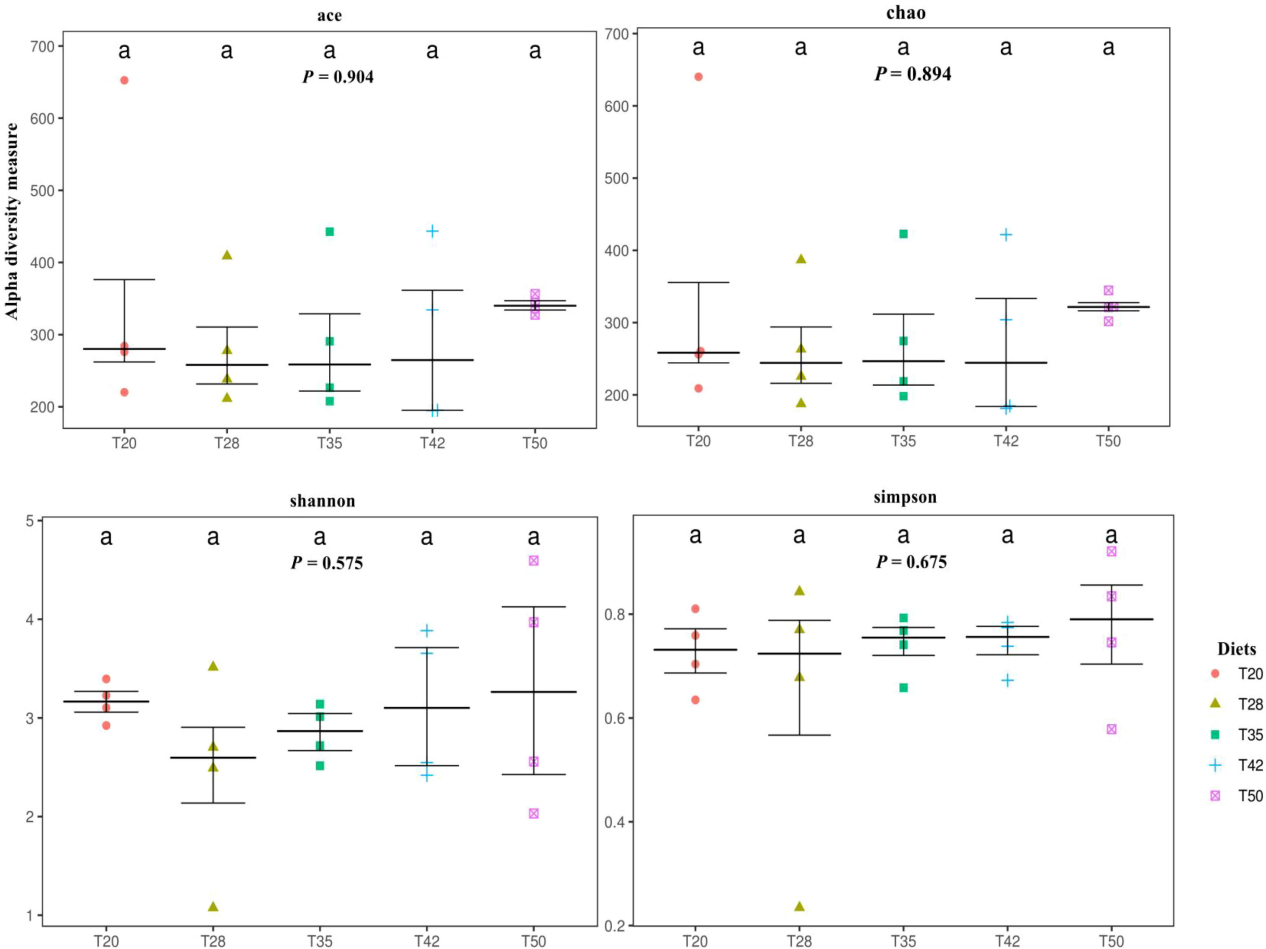
Alpha diversity of bacterial communities. Each panel represents one alpha diversity measure. Abundance-based coverage estimator (ACE) and Chao indices are richness estimators to estimate the total number of operational taxonomic units (OTUs) present in a community. Shannon and Simpson indices are indicators of microbial diversity. The proportion of soybean meal in the diets was 20% (T20), 28% (T28), 35% (T35), 42% (T42), or 50% (T50).

**Figure 7:**
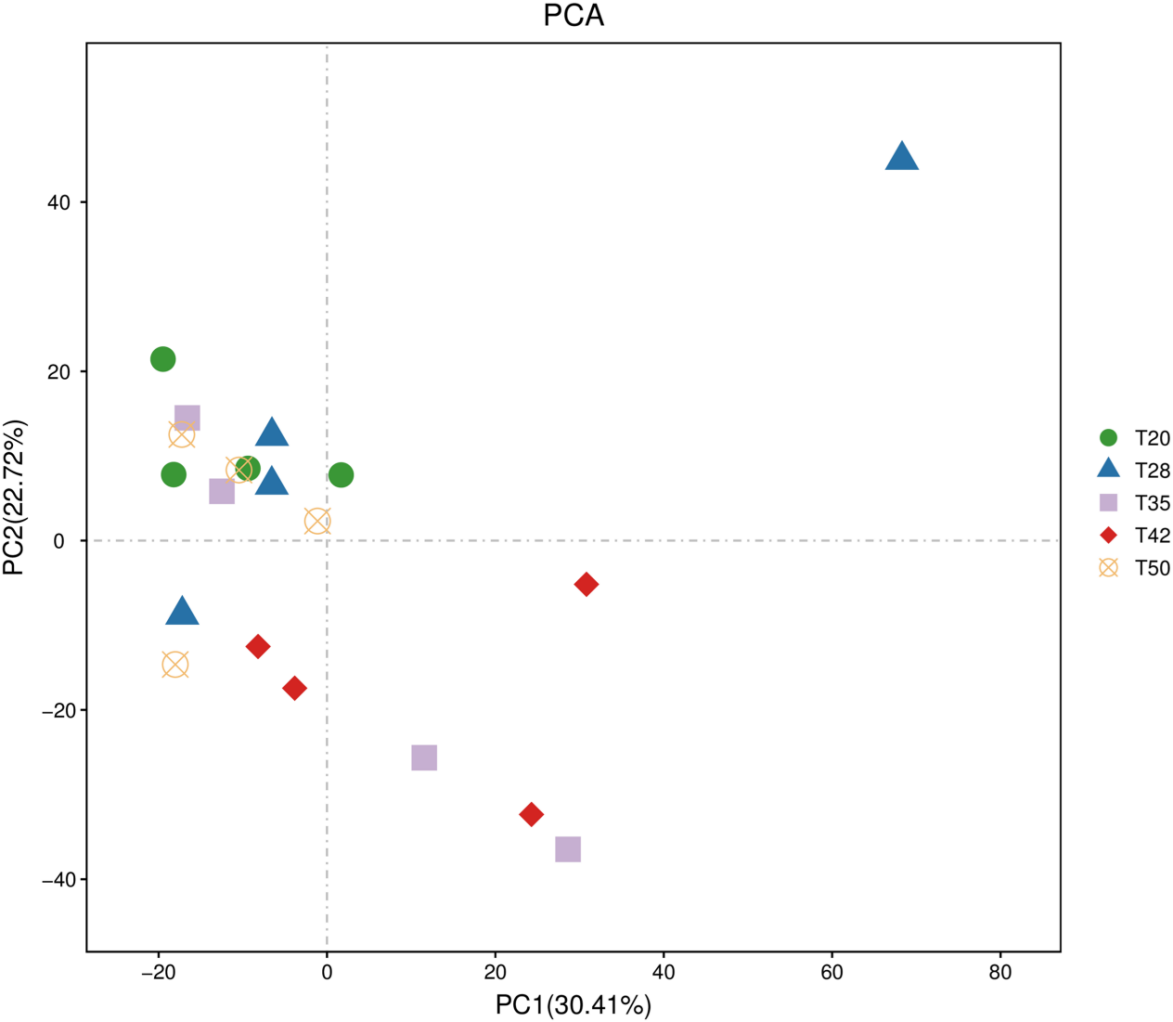
Beta diversity of bacterial communities. The operational taxonomic unit (OTU) principal component analysis (PCA) plots. The proportion of soybean meal in each diet was 20% (T20), 28% (T28), 35% (T35), 42% (T42), or 50% (T50).

**Figure 8:**
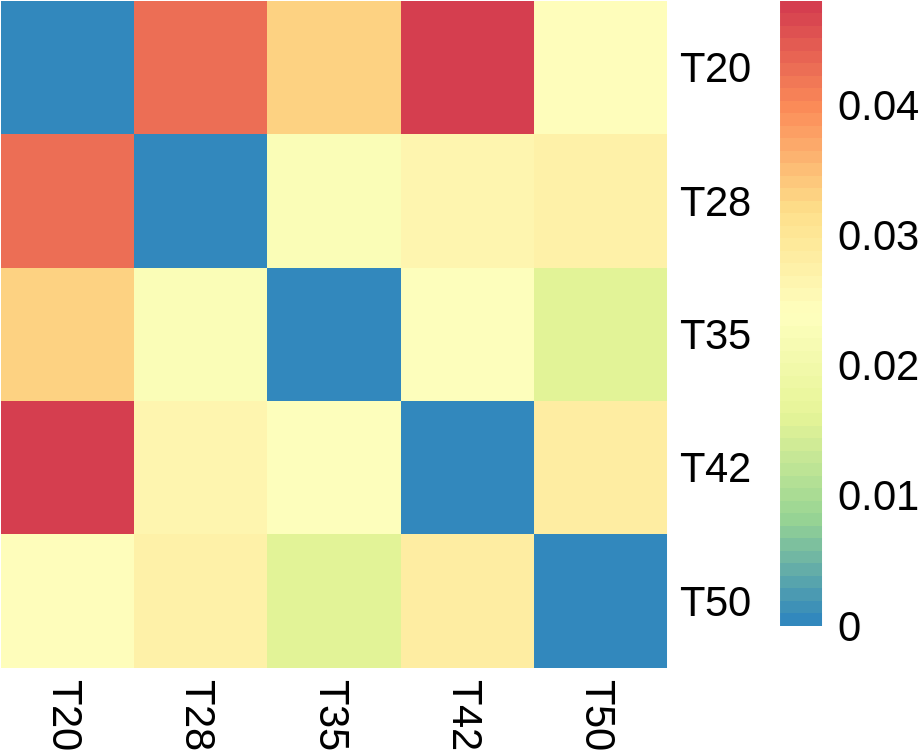
Beta diversity of bacterial communities. Heatmap analysis using the weighted UniFrac distances. The proportion of soybean meal in each diet was 20% (T20),28% (T28), 35% (T35), 42% (T42), or 50% (T50).

The abundance-based coverage estimator (ACE), Chao, Shannon, and Simpson indicators, measures of alpha diversity, were similar (*p* > 0.05) among groups (**Figure 6**). In the case of beta diversity, distinction between bacterial communities was low, as most of the plots form separate clusters within each group (**Figure 7**). Similarly, when the beta diversity of bacterial communities was evaluated by the weighted UniFrac distances, a heatmap analysis showed minimal difference among groups (**Figure 8**).

**Figure 9:**
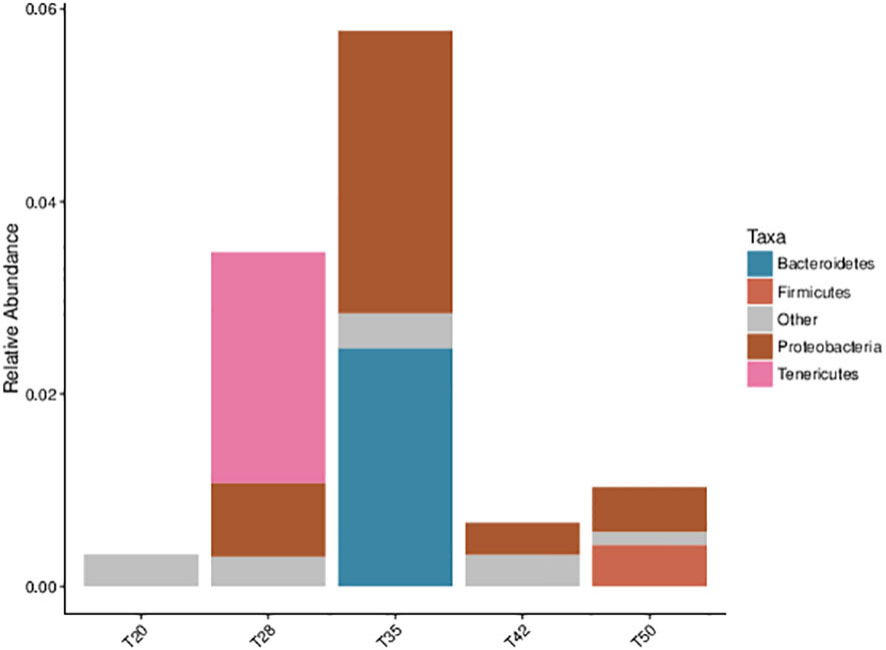
Phenotypic classification prediction of pathogenic bacterial communities using BugBase based on the Greengenes database. The contribution (relative abundance) of bacterial communities at phylum level to the phenotypic function. The proportion of soybean meal in each diet was 20% (T20), 28% (T28), 35% (T35), 42% (T42), or 50% (T50).

The contribution of bacterial communities at a phylum level to the phenotypic function (pathogenic) is shown in **Figure 9**. In contrast with T20, the major pathogenic bacterial communities in other groups were Proteobacteria, Tenericutes, Bacteroidetes, and Firmicutes.

## Discussion

### Serum metabolites

Serum metabolites generally reflect the physiological status, health level, and nutrient metabolism of aquatic animals. In this study, partial changes in serum metabolites suggest that the supplementation of shrimp diets with soybean meal adversely impacted these metabolite profiles. As part of the immune system, concentrations of serum GLOB can reflect the inflammation and nutritional status of animals. Generally, the serum concentration of GLOB is positively correlated with inflammation level (Bu et al., 2018). Therefore, the increased GLOB concentration in the serum of shrimp fed soybean meal-supplemented diets in this study indicates that dietary soybean meal may induce inflammation and thus interfere with the immune system of shrimp. A similar result was also reported by Chen et al. (2014), who found that the inclusion of 60% soybean meal in the diets of grass carp increased their serum GLOB concentration. Both ALT and AST are related to liver damage or necrosis when their measured activity increases in blood, because the increased permeability of the cell membrane induced by injury or stress causes ALT and AST to be released from cells into the blood more rapidly *via* the cell membrane barrier (Peng et al., 2021b). In this study, the increased serum ALT and AST activities of *L. vannamei* suggest that dietary supplementation with soybean meal may impair the hepatopancreatic health of shrimp. This is consistent with the histological observation of the hepatopancreas in this study, which found that the degeneration of or damage to hepatocytes increased as the dietary levels of soybean meal increased. Liver injury is also commonly observed in fish when fish meal is replaced by soybean meal (Hernández et al., 2007; Lim et al., 2011; Hu et al., 2018). HDLC, an anti-atherosclerosis lipoprotein, transports cholesterol from other tissues to the liver for metabolism, preventing cholesterol from being deposited in blood vessel walls. Therefore, a low serum HDLC concentration normally indicates an increased risk of atherosclerosis, heart disease, and other conditions. In this study, supplementation of *L. vannamei* diets with soybean meal linearly decreased serum HDLC concentration, suggesting that soybean meal may cause dyslipidemia in shrimp. Similarly, Liu et al. (2010) documented that replacing dietary fish meal with soybean meal decreased serum HDLC concentration in Japanese flounder (*Paralichthys olivaceus*).

### Antioxidant and immune response

The increase in serum TAOC as dietary levels of soybean meal increased in this study suggests that supplementation with soybean meal enhanced the antioxidant capacity of shrimp. This is also reflected in the increased serum CAT activity of shrimp. The results of this study are consistent with the finding by Li et al. (2020) that a diet in which fish meal was replaced with fermented soybean meal increased TAOC and MDA concentrations in the serum of *Anguilla japonica*. It has been reported that excessive accumulation of MDA can lead to oxidative damage (Ding et al., 2015). In this study, the increased antioxidant capacity and MDA concentrations in *L. vannamei* were probably a response to the oxidative damage stimulated by the supplementation of diets with soybean meal. An et al. (2018) reported that replacing more than 20% of fish meal with soybean meal in diets increased serum antioxidant capacity and caused oxidative stress in *Epinephelus coioides*.

The results of this study indicate that supplementation with soybean meal in shrimp diets increases serum NOS and PPO activity in shrimp. Commonly, NOS is a key rate-limiting factor in the synthesis of nitric oxide, which is a major catalyst of phagocytosis in the immune system (Ignarro, 1996). PPO is the product of the prophenoloxidase activation system of crustaceans, which plays an important role in recognizing foreign bodies, releasing opsonins to promote phagocytosis, and producing lectins or lysozyme to exclude foreign bodies (Liu et al., 2005). Therefore, the expression of NOS and PPO is generally regarded as an indication of immunostimulation or an increased risk of stress. In this study, the increase in serum NOS and PPO activity as dietary levels of soybean meal increased may be attributed to immunostimulation or stress induced by soybean meal in shrimp diets. Similarly, Wu et al. (2008) reported that cadmium stress induced an increase in nitric oxide synthase activity in the serum of *L. vannamei*. Peng et al. (2015) documented that replacing 6% of fish meal with fermented soybean meal in *L. vannamei* diets increased the PPO activity in the hemolymph of the shrimp.

### Histological appearance of hepatopancreas

Histomorphology is an important part of understanding liver pathology. The observation of hepatopancreas histomorphology in this study suggested that dietary supplementation with soybean meal caused lesions in the hepatopancreas of *L. vannamei*. This may be due to the presence of antinutritional factors in soybean meal. To the best of our knowledge, this is the first study to evaluate the effects of dietary supplementation with soybean meal on the hepatopancreas histomorphology of *L. vannamei*. Chen et al. (2014) reported that a diet comprising 60% soybean meal caused liver damage in grass carp (*Ctenopharyngodon idellus*), as demonstrated by abnormal nuclear morphology, increased cell area and lipid content, and aggravated vacuolation. Similar results were also observed by Zhang et al. (2012) in the large yellow croaker (*Pseudosciaena crocea*): replacing 45% of fish meal with soybean meal decreased hepatocyte numbers and increased hepatocyte vacuolation. A number of studies have suggested that supplementation with plant protein sources in diets could impair liver function and induce liver damage in aquatic animals, as shown by reduced lipoprotein synthesis and the accumulation of lipids in the liver (Robaina et al., 1995; Feng et al., 2016; Hu et al., 2018; Lin et al., 2019; Chen et al., 2021).

### Bacterial microbiota

Although the effects of dietary soybean meal on the growth and physiology of *L. vannamei* have been well evaluated, little information is available on the response of intestinal bacterial microbiota. It has been reported that the intestinal bacterial microbiota is closely related to the intestinal health of shrimp (Zhao et al., 2021b). In this study, within the phyla identified, over 94% of the sequences were classified within the phyla Proteobacteria, Tenericutes, Firmicutes, and Bacteroidetes. These dominant bacterial phyla identified in the intestine of *L. vannamei* were consistent with previous studies (Li et al., 2007; Wu et al., 2016; Shao, 2019; Zhao et al., 2021b), suggesting that the composition of the intestinal bacterial microbiota of *L. vannamei* was conserved to some extent. Among the dominant bacterial phyla, bacteria of the phylum Proteobacteria were the most abundant in the intestine of *L. vannamei*. This finding is similar to other reports (Cornejo-Granados et al., 2017; Hou et al., 2018; Shao, 2019). Furthermore, this study showed that supplementation with soybean meal in shrimp diets increased the abundance of Proteobacteria and Cyanobacteria but decreased the abundance of Tenericutes. Similarly, Shao (2019) reported that the replacement of fish meal with fermented soybean meal in *L. vannamei* diets increased the abundance of Proteobacteria, but decreased the abundance of Tenericutes in the intestine of shrimp. Zhao et al. (2021b) documented that dietary inclusion of soybean meal increased the abundance of Cyanobacteria in the intestinal bacterial microbiota of *L. vannamei*.

At the genus level, the composition of bacterial microbiota in the intestine of *L. vannamei* has been identified, and the effects of certain feedstuffs and additives on the bacterial genera found in the intestine of *L. vannamei* have been reported (Wu et al., 2016; Tang et al., 2021; Wu et al., 2021b; Zhu et al., 2022). However, information on the response of the intestinal bacterial genera of *L. vannamei* to dietary supplementation with soybean meal is scarce. Zhao et al. (2021b) evaluated the effects of replacing fish meal with soybean meal on the composition of intestinal microflora of *L. vannamei* only at the phylum and family levels. Although Shao (2019) reported the composition of the intestinal bacterial genera of *L. vannamei* fed with fermented soybean meal, the genera with higher abundance were mostly from unidentified families. Therefore, the 10 most abundant bacterial genera initially identified in the intestine of *L. vannamei* in this study provide the reference for the subsequent evaluation of the intestinal health of shrimp fed diets supplemented with soybean meal.

Alpha diversity refers to diversity within a certain biotope and is commonly described using specific alpha diversity indices such as ACE, Chao, Shannon, and Simpson (Peng et al., 2022b). This study shows that dietary treatments did not affect the ACE, Chao, Shannon, and Simpson indices of *L. vannamei*, suggesting that supplementation of *L. vannamei* diets with soybean meal had no significant effect on the alpha diversity of bacterial microbiota in the intestine of shrimp. Similar results were reported by Zhao et al. (2021b), who found that replacing 50% of fish meal with soybean meal in the diets of *L. vannamei* had no significant influence on the alpha diversity as measured with the Chao, Shannon, and Simpson indices. Shao (2019) also reported that replacing up to 40% of fish meal with fermented soybean meal in *L. vannamei* diets did not alter the alpha diversity as measured with the Shao, Shannon, and Simpson indices.

Beta diversity reveals differences in the bacterial community structure and is commonly assessed using the weighted UniFrac distances (Peng et al., 2018) and visualized as principal component analysis plots (Yang et al., 2018). In this study, dietary treatments had no significant influence on the beta diversity of the intestinal bacterial microbiota of *L. vannamei*. This is consistent with the observation by Shao (2019) that replacing dietary fish meal with fermented soybean meal in *L. vannamei* diets did not alter the beta diversity of intestinal bacterial communities.

Several large microbial pathway databases, such as the Kyoto Encyclopedia of Genes and Genomes (Kanehisa et al., 2012) and Clusters of Orthologous Groups (Tatusov et al., 1997), can be used to analyze the functionality of a microbiome. However, current tools cannot use sequencing data to readily infer common microbial phenotypes. Although the function prediction of intestinal bacterial microbiota has been reported in *L. vannamei* (Yu et al., 2018; Shao, 2019), the phenotype analysis of intestinal bacterial microbiota is limited. This is the first study to assess the phenotypes of intestinal bacterial microbiota impacted by soybean meal in *L. vannamei* diets. BugBase software (a microbiome analysis tool that determines high-level phenotypes present in microbiome samples) is usually used to classify and compare seven phenotypes of intestinal flora: Gram positive, Gram negative, biofilm forming, pathogenic, mobile element containing, oxygen utilizing, and oxidative stress tolerant (Ward et al., 2017; Yi et al., 2021). In this study, the phenotypic classification prediction of pathogenic bacterial communities in the intestine of *L. vannamei* fed soybean meal-containing diets suggested that the phylum Proteobacteria plays a major role in contributing to pathogenic phenotype, followed by Tenericutes, Bacteroidetes, and Firmicutes. BugBase predicted that *L. vannamei* fed a diet supplemented with soybean meal, compared with shrimp fed a control diet, would contain significantly more potentially pathogenic bacteria in their intestine. Sun (2015) reported that replacing 50% of fish meal with soybean meal in *L. vannamei* diets induced injury of the intestinal structure and increased the abundance of pathogenic bacteria. A similar result was also observed by Zhao et al. (2021b) in *L. vannamei* and by Yin (2019) in grouper (*Epinephelus fuscoguttatus* ♀ × *E. lanceolatu* ♂).

## Conclusion

Supplementation of *L. vannamei* diets with soybean meal induced varying degrees of inflammation and injury of the hepatopancreas, as shown by the assessment of serum metabolites and hepatopancreas histomorphology. An increase in serum antioxidant capacity and the immunity of shrimp is probably in response to the oxidative damage stimulated by dietary supplementation with soybean meal. Although inclusion of soybean meal in *L. vannamei* diets significantly altered the relative abundances of intestinal bacterial microbiota at the phylum level, it had a minimal impact on the top 10 bacterial genera and had no significant influence on bacterial diversity. Phenotype analysis suggested that dietary supplementation with soybean meal led to significantly more potentially pathogenic bacteria in the intestine of *L. vannamei*.

## Data availability statement

The data sets presented in this study can be found in online repositories. The names of the repository/repositories and accession number(s) can be found in the article/supplementary material.

## Author contributions

KP and WH conceived the experiment. KP, JQ, CL, HL, ZL, and DL carried out the experiment. KP analyzed the data and wrote the paper. All authors contributed to the article and approved the submitted version.
